# Alteration in plasma free amino acid levels and its association with gout

**DOI:** 10.1186/s12199-017-0609-8

**Published:** 2017-03-16

**Authors:** MH Mahbub, Natsu Yamaguchi, Hidekazu Takahashi, Ryosuke Hase, Hiroki Amano, Mikiko Kobayashi-Miura, Hideyuki Kanda, Yasuyuki Fujita, Hiroshi Yamamoto, Mai Yamamoto, Shinya Kikuchi, Atsuko Ikeda, Naoko Kageyama, Mina Nakamura, Yasutaka Ishimaru, Hiroshi Sunagawa, Tsuyoshi Tanabe

**Affiliations:** 1 Department of Public Health and Preventive Medicine, Yamaguchi University Graduate School of Medicine, 1-1-1 Minami-Kogushi, Ube, Yamaguchi 755-8505 Japan; 2 Division of Health Administration and Promotion, Graduate School of Medicine, Tottori University, Yonago, Japan; 3 Department of Biochemistry, Shimane University Faculty of Medicine, Izumo, Japan; 4 Department of Environmental Medicine and Public Health, Shimane University Faculty of Medicine, Izumo, Japan; 5 Institute for Innovation, Ajinomoto Co. Inc, Kawasaki, Japan

**Keywords:** Amino acids, Plasma, Profile, Gout, Relationship

## Abstract

**Background:**

Studies on the association of plasma-free amino acids with gout are very limited and produced conflicting results. Therefore, we sought to explore and characterize the plasma-free amino acid (PFAA) profile in patients with gout and evaluate its association with the latter.

**Methods:**

Data from a total of 819 subjects (including 34 patients with gout) undergoing an annual health examination program in Shimane, Japan were considered for this study. Venous blood samples were collected from the subjects and concentrations of 19 plasma amino acids were determined by high-performance liquid chromatography–electrospray ionization–mass spectrometry. Student’s *t-*test was applied for comparison of variables between patient and control groups. The relationships between the presence or absence of gout and individual amino acids were investigated by logistic regression analysis controlling for the effects of potential demographic confounders.

**Results:**

Among 19 amino acids, the levels of 10 amino acids (alanine, glycine, isoleucine, leucine, methionine, phenylalanine, proline, serine, tryptophan, valine) differed significantly (*P* < .001 to .05) between the patient and control groups. Univariate logistic regression analysis revealed that plasma levels of alanine, isoleucine, leucine, phenylalanine, tryptophan and valine had significant positive associations (*P* < .005 to .05) whereas glycine and serine had significant inverse association (*P* < .05) with gout.

**Conclusions:**

The observed significant changes in PFAA profiles may have important implications for improving our understanding of pathophysiology, diagnosis and prevention of gout. The findings of this study need further confirmation in future large-scale studies involving a larger number of patients with gout.

## Background

Gout, the most prevalent inflammatory joint disease is usually characterized by recurrent attacks of intense pain. It is predominant in men and also in older women [1]. Currently, the global prevalence of gout is estimated to be at least 1–2% in the general population and 3–4% in the adult population [2]. The prevalence can be as high as 7% among the male population over 75 years of age [3]. In Japan, the overall prevalence of gout was found to be lower in a survey which was 0.51% overall and 1.1% amongst men [4]. The incidence and prevalence of gout are found to be increasing in many parts of the world [5, 6]. With the aging of world population, the global burden of gout continues to rise [7]. Therefore, better understanding of various factors and mechanisms underlying the pathophysiology of gout is necessary for the proper diagnosis and management of it.

The most important underlying mechanism in gout involves elevated levels of uric acid in the blood. Such a persistent increase in the levels of serum uric acid causes crystallization of it and intra-articular formation and deposition of monosodium urate (MSU) crystals which can trigger the painful attacks of gout [8]. The serum uric acid is the end product of an exogenous (from food) pool of purines and endogenous (from liver, intestines, muscles, kidneys and the vascular endothelium) purine metabolism [9]. Certain amino acids take part in the biosynthesis of purine and subsequent formation of uric acids. For example, amino acids like glutamine, glycine, and serine are utilized in increased amounts for the formation of uric acid in gout [10]. Therefore, it is reasonable to postulate that amino acids play important roles in the pathogenesis of gout.

Plasma free amino acids (PFAAs) abundantly circulate as a medium linking all organ systems in the human body; the PFAA profiles have been shown to be influenced by metabolic variations in specific organ systems induced by specific diseases [11]. PFAA profiles can be used as reliable markers for monitoring the risks of various diseases in various populations and also the improvements in physiological states [12–14]. Therefore, amino acid profiles are increasingly being used in the evaluation of various diseases. Analysis of PFAA profiles with a high degree of validity and reliability can be useful in understanding the underlying pathophysiology and assessing the severity of a disorder. Furthermore, various indexes developed from PFAA profiles have shown the potential for diagnosis of various pathological conditions [11, 14, 15]. Understanding the variations in PFAA profiles among various populations including patients with gout seems to be important which may guide to the diagnosis of gout. However, the number of studies investigating the changes in PFAA profiles associated with gout is very limited. Also, there is a lack of published research works, especially in recent times, on the relationship between the amino acid profiles and gout. A few previous studies studies reported altered PFAA profiles in patients with gout. Compared to control subjects, some researchers reported a significant increase for some of the amino acids and a significant decrease for the others in patients with gout [10, 16], whereas other researchers reported hyperaminoacidemia for all the investigated amino acids in the patients or similar amino acid spectrums in normal subjects and patients with gout [17, 18]. As understandable, the findings are conflicting as the observed changes in those studies are inconsistent for various amino acids. Furthermore, those studies aimed at finding the significant group differences between gout patients and control subjects in the concentrations of various amino acids, and did not investigate the relationship of amino acids with gout. Therefore, the purpose of the present study was to further explore and characterize the PFAA profiles in patients with gout and evaluate its association with the latter.

## Methods

A total of 831 subjects who underwent their annual health check-up between June and July 2012 at different health examination centers in Shimane Prefecture, Japan and for whom workplace health examination was not applicable, were considered for inclusion in this study. The health examination included physical examination, clinical and laboratory tests, and a self-administered questionnaire containing personal and medical history. Based on the questionnaire data, 12 subjects were excluded from the study due to lack of information on gout. Finally, 34 subjects were identified as having an established diagnosis of gout and were treated as the patient population, and the rest 785 subjects were treated as the control population in this study. Among the patients, 26 were currently taking medications for gout and the rest 8, currently without any medications for it. The subjects had no serious health problems like cancer or renal failure.

An oral explanation of the study protocol was made in detail to the study participants and written informed consent was obtained from all of them. The current study protocol was approved by the institutional review board of Shimane University Hospital (No. H25-26-2).

### Measurement of plasma amino acid concentrations

Five ml of blood samples were collected and analyzed for plasma amino acid concentrations following the protocol previously described elsewhere [11, 19–21]. Briefly, after overnight fasting, venous blood samples were collected from the cubital vein of the seated subjects in tubes which contained ethylenediaminetetraacetic acid (EDTA; Termo, Tokyo, Japan). The tubes were placed on ice immediately and kept there for about 15 min. After centrifugation of tubes under 4 °C at 3,000 rpm for 15 min, the plasma was immediately separated into tubes and stored at −80 °C. The tubes were kept there until (within 2 weeks to 2 months) the desired analysis for plasma amino acids. The plasma samples were deproteinized using acetonitrile at a final concentration of 80% before measurements. The amino acid concentrations in the plasma were measured by high-performance liquid chromatography–electrospray ionization–mass spectrometry (HPLC–ESI–MS) followed by precolumn derivatization which allows such measurements with high accuracy. PFAA profiling included the measurement of absolute concentrations (in μmol/L) of the following 19 amino acids: alanine (Ala), arginine (Arg), asparagine (Asn), Citrulline (Cit), glutamine (Gln), glycine (Gly), histidine (His), isoleucine (Ile), leucine (Leu), lysine (Lys), methionine (Met), ornithine (Orn), phenylalanine (Phe), proline (Pro), serine (Ser), threonine (Thr), tryptophan (Trp), tyrosine (Tyr), and valine (Val).

### Statistical analyses

The continuous variables in this study showed a non-normal distribution by Kolmogorov-Smirnov and Shapiro-Wilk tests. Hence, statistical analyses were performed with those variables after logarithmic transformation of data. The continuous variables were expressed as geometric mean and its 95% confidence interval (95% CI). Smoking status was categorized as current/past smokers and non-smokers, and alcohol consumption, as current drinkers and non/rare-drinkers. For convenience of graphical presentation of individual amino acids, they were divided into 3 groups according to their plasma geometric concentrations. The differences between the patient and control groups were assessed with Student’s *t-*test and Chi-square (*χ* ^2^) test for continuous and categorical variables, respectively. To further explore the amino acids associated with the outcome (gout), logistic regression analysis was carried out with the individual amino acids that did differ between the two groups at *P <* .05. The models were adjusted for the potential confounding demographic factors and corresponding odds ratios (OR), 95% confidence interval (CI) and *P*-values were obtained. All statistical tests were considered as two-tailed, and the significance level was set at the value of *P <* .05. The software package SPSS version 22 for Windows (SPSS Inc., Chicago, IL, USA) was used to perform the statistical analyses.

## Results

The demographic and clinical characteristics of the current study subjects have been presented in table 1. Most study participants in both patient and control groups were elderly for whom smoking and drinking were common. Statistically significant differences between the patient and control groups were observed for gender, BMI, and smoking and drinking status (*P* < .001 to .01). Furthermore, significant differences in terms of alanine aminotransferase/ALT, gamma-glutamyl transpeptidase/γ-GTP, high-density lipoprotein cholesterol/HDLC, and triglycerides/TG (*P* < .001 to .05) were also observed between the two groups. Among the participants of this study, the commonly used medications were for hypertension (patients, 20/34 or 58.8%; controls, 329/785 or 41.9%), diabetes mellitus (patients, 5/34 or 14.7%; controls, 75/785 or 9.6%), and dyslipidemia (patients, 10/34 or 29.4%; controls, 272/785 or 34.6%). With respect to the use of medications, the patients and controls did not differ significantly for diabetes mellitus (*χ* ^2^ = .981; *P* = .322), dyslipidemia (*χ* ^2^ = .396; *P* = .585), or for hypertension (*χ* ^2^ = 3.812; *P* = .075).

**Table 1:** Demographic and clinical characteristics of study subjects. Values are expressed as geometric mean and 95%CI for continuous variables, and number (*n*) and percent (%) for categorical variables

Characteristics	Patient (*n* = 34)		Control (*n* = 785)		*P*-value^*^
	Geometric mean or n	95%CI or %	Geometric mean or n	95%CI or %	
Age (years)	72.9	70.3–75.7	72.1	72.1–73.4	.918
Gender					<**.001**
Male	29	85.3	298	38	
Female	5	14.7	487	62	
BMI (kg/m^2^)	24.0	23.0–25.1	22.1	21.8–22.3	**.004**
Smoking status					**< .001**
Non-smokers	17	50	619	78.9	
Smokers	17	50	166	21.1	
Alcohol					**.008**
Non-drinkers	17	50	558	71.1	
Drinkers	17	50	227	28.9	
SBP (mmHg)	127.3	122.2–132.6	127.4	126.2–128.5	.994
DBP (mmHg)	73.5	70.5–76.6	72.7	72.0–73.3	.603
AST (IU/l)	26.9	23.2–31.3	23.5	23.0–23.9	.067
ALT (IU/l)	21.6	18.1–25.9	16.5	16.1–17.0	**.005**
γ-GTP (IU/l)	35.7	24.7–51.6	21.7	20.8–22.6	**.010**
Glucose ^a^ (mg/dl)	96.2	90.3–102.4	90.4	89.6–91.2	.054
HbA1C ^b^(%)	5.6	5.4–5.9	5.5	5.5–5.5 ^c^	.122
HDLC (mg/dl)	51.1	46.3–56.3	61.8	60.7–62.9	**< .001**
LDLC (mg/dl)	110.9	103.9–118.4	117.2	115.3–119.0	.161
TG (mg/dl)	115.2	91.3–145.2	90.6	88.1–93.3	**.045**

^*^Bold values indicate statistically significant difference

*ALT*, alanine aminotransferase, *AST*, aspartate aminotransferase, *BMI* body mass index, *DBP* diastolic blood pressure, *γ-GTP* gamma-glutamyl transpeptidase; *Glucose* fasting blood glucose, *HbA1C* haemoglobin A1C, *HDLC* high-density lipoprotein cholesterol, *LDLC* low-density lipoprotein cholesterol, *SBP* systolic blood pressure, *TG* triglycerides

^a^ Glucose: 4 missing values for control group

^b^ HbA1C: 1 missing value for patient group and 17, for control group

^c^ 5.47–5.53

Data on plasma uric acid were available only for a limited number of subjects: 19 (19/34 or 55.9%) patients and 392 (392/785 or 49.9%) control subjects. Overall, the concentration of uric acid was higher in the patients compared to the controls which also significantly differed between the two groups (geometric mean and 95%CI for patients and control groups were 6.2 mg/dl and 5.7–6.7 mg/dl, and 4.9 and 4.7–5.0 mg/dl, respectively; *P* < .001 by Student’s t-test).

**Fig. 1:**
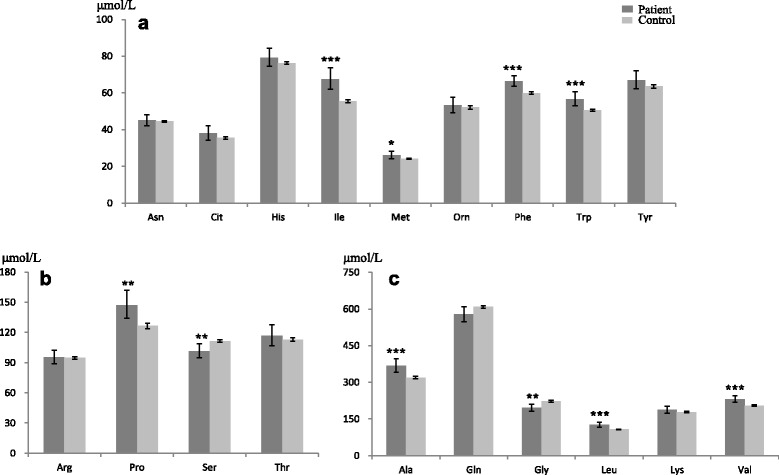
Comparison between gout patients (*n* = 34) and control subjects (*n* = 784; 1 missing value) for individual amino acids: **a**, amino acids with mean concentration <90 μmol/L; **b**, amino acids with mean concentration between 90 and 150 μmol/L; **c**, amino acids with mean concentration >150 μmol/L. Values are shown as geometric mean and 95% CI. Significantly different from the corresponding control value: ^*^ *P* < .05, ^**^ *P* < .005 and ^***^ *P* < .001

Figure 1 shows the concentrations of the individual amino acids for the two groups. Significant differences between patients and controls were found for the plasma levels of Ala, Gly Ile, Leu, Met, Phe, Pro, Ser, Trp, and Val (*P* < .001 to .05). Compared to the corresponding values in the control subjects, the concentrations of Ala, Ile, Leu, Met, Phe, Pro, Trp and Val were significantly higher in the plasma samples of the patients with gout; in contrast, the concentrations of Gly and Ser were significantly lower in the patients.

To identify the association between the selected amino acid levels and gout, we performed logistic regression analysis without and with adjustment for the demographic factors that significantly differed between the patient and control groups (as in table 1). Before adjustment, all the amino acids that significantly differed between the two groups showed their association with gout. When the logistic regression model was adjusted for 3 demographic factors (BMI, smoking status, and alcohol consumption), all the amino acids retained their association with gout except for Met and Pro. Among them, plasma levels of Ala, Ile, Leu, Phe, Trp and Val had significant positive associations [OR between 1.50 and 1.78; 95% CI between 1.02 and 1.25 (lower) and 2.20 to 2.53 (upper); *P* < .005 to .05)], whereas Gly (OR 0.60; 95% CI 0.38 to 0.94; *P* < .05) and Ser (OR 0.67; 95% CI 0.48 to 0.95; *P* < .05) had significant inverse association with gout (Table 2). However, when this model was further adjusted for gender, the association was just significant for plasma levels of Ile (*P* = .049) and Ser (*P* = .048) and remarkable for Gly (*P* = .061) and Phe (*P* = .050) (Table 2).

**Table 2:** Logistic regression analysis for association between gout and individual amino acids without and with adjustment for potential confounding demographic factors. The odds ratios were estimated per one SD change in the concentrations of corresponding amino acids

	Model I ^a^				Model II ^b^				Model III ^c^			
Amino acid	OR	95% CI		*P*-value^*^	OR	95% CI		*P*-value^*^	OR	95% CI		*P*-value^*^
		Lower	Upper			Lower	Upper			Lower	Upper	
Ala	1.90	1.34	2.68	**<.001**	1.52	1.03	2.23	**.035**	1.31	0.88	1.95	.183
Gly	0.52	0.34	0.81	**.003**	0.60	0.38	0.94	**.025**	0.64	0.40	1.02	.061
Ile	2.16	1.57	2.97	**<.001**	1.78	1.25	2.53	**.001**	1.45	1.00 ^**d**^	2.10	**.049**
Leu	2.14	1.53	2.99	**<.001**	1.69	1.17	2.44	**.005**	1.35	0.92	1.99	.127
Met	1.45	1.05	2.00	**.026**	1.13	0.79	1.62	.502	0.89	0.61	1.31	.571
Phe	1.86	1.37	2.54	**<.001**	1.60	1.14	2.26	**.007**	1.44	1.00 ^**d**^	2.06	.050
Pro	1.59	1.16	2.17	**.004**	1.35	0.94	1.93	.108	1.06	0.70	1.60	.792
Ser	0.60	0.43	0.84	**.003**	0.67	0.48	0.95	**.025**	0.71	0.50	1.00	**.048**
Trp	1.99	1.37	2.88	**<.001**	1.53	1.03	2.26	**.033**	1.28	0.86	1.90	.221
Val	1.92	1.36	2.72	**<.001**	1.50	1.02	2.20	**.037**	1.20	0.80	1.80	.385

^*^Bold *P*-values indicate statistically significant level of association with CI not including 1

^a^ Model I: without adjustment

^b^Model II: adjusted for BMI, smoking status, and alcohol consumption

^c^Model III: adjusted for gender, BMI, smoking status, and alcohol consumption

^d^Rounded to 1

## Discussion

Plasma free amino acids play important physiological roles in the biosynthesis and catabolism of various metabolites and regulators of many metabolic pathways [11]. A disease state causes specific metabolic changes and subsequent alterations in PFAA profiles in the human body. In this study, we measured the plasma concentrations of 19 amino acids by using the advanced HPLC-ESI-MS technique and investigated the possible association between PFAAs and gout that has not been reported yet.

As revealed in our study, there were significant elevations in the levels of ALT, γ-GTP and TG levels, and a significantly lower HDLC level in patients, compared with the control subjects. These findings are in line with the existing literature as gout is associated with increased levels of uric acid in the blood, and hyperuricemic men and women have shown the coexistence of hypertriglyceridemia, hypercholesterolemia, and hypo-HDLC [22]; and an increased level γ-GTP might be associated with the latter [23]. In a recent study, Chen et al. found a significant association between hyperuricemia and ALT elevation [24]. The authors postulated that an increased oxidative stress in hyperuricemia might be the underlying cause for such an elevation in ALT levels; moreover, it may also stimulate the synthesis of γ-GTP in hyperuricemic subjects [23, 24].

In this study, we observed significantly different patterns in the levels of a number of amino acid between the patients with gout and control subjects. Our results correspond to the findings from the previous research works, in which a number of amino acids in serum or plasma of gout patients and control subjects have been measured and compared which indicated varying patterns of amino acid concentrations among the participants. Our findings are consistent with those of Kaplan et al. [18], who found significantly elevated levels of serum Ala, Ile and Leu, Val, Tyr, Phe and Lys in patients with gout. In a study, Yü et al. [16] compared PFAA concentrations in male patients with primary gout with those of control males, and found significant (*P* < .05 to .01) increases in Ala and Ile in the patients as also observed in our study. Similarly, in another study including a small number of subjects (7 patients with primary gout and 6 control males), Yü et al. [10] observed a significantly (*P* = 0.01) elevated level of only Ile and a remarkable (*P* = 0.05) increase in Leu in the patients.

In this study, compared to the control subjects, we observed lower levels of Gly and Ser among the patients with gout, which is also supported by the study of Yü et al. [16], who found significant (*P* < .05 to .01) decreases in the mean concentrations of Gly and Ser among such patients. However, in the other study by Yü et al. [10], the level of depression in Gly was just significant (*P* = 0.05). On the other hand, Kaplan et al. [18] observed a significant increase in serum Gly level among such patients. Similar to the observation in our study, the concentration of plasma Gln was found to be normal among the patients in a study conducted by Pagliara and Goodman [25]. In contrast to the findings of all the above-mentioned studies, Derrick and Hanley [17] did not reveal any significant differences in amino acid profiles between gout patients and normal control subjects.

The overall findings for the trend of differences in various amino acid levels between gout patients and control subjects were more or less similar across studies except the study of Kaplan et al. [18] for the level of serum Gly. However, considering the relevant changes in amino acid concentrations in different studies including the current one, we postulate that the amino acid profile is altered in gout causing an elevation in a number of amino acid concentrations and a depression in others. Gly and Ser play important roles in the biosynthesis of purine; they are the precursors of uric acid. Lower concentrations of these amino acids in patients with gout is probably due to the fact that they donate either amide nitrogen or carbon or both to the purine ring which are utilized in increasing amounts for the formation of uric acid in gout [16]. Furthermore, there is a possibility that the alterations in plasma amino acid balance caused by deficiency of particular amino acids in the plasma due to their involvement in purine biosynthesis affect the plasma amino acid profile and lead to the increased level of some other amino acids as observed in our study.

Plasma amino acids are highly correlated with each other. Armstrong and Stave [26] in their study observed high interrelations among a large group of amino acids in healthy children and adults. Also, in this study, we observed significant correlations between the levels of selective amino acids in both patient and control groups (results not shown). Therefore, instead of multivariate analysis, we performed univariate logistic regression analysis to explore the association between selective individual amino acids with gout. The logistic regression model with adjustment for BMI, smoking status and alcohol consumption revealed both positive and negative association between specific amino acids and gout. As those amino acids lost their significance or some remained marginally significant for the association with gout after further adjustment of the model for the variable gender, this might have been caused by the fact that a small number of female patients with gout could be included in this study. Nonetheless, as we believe, the role of those amino acids in gout should not be underestimated as they showed highly significant differences between the patient and control groups in this study and also in a number of studies as discussed earlier. However, direct comparison of the current findings for the association between amino acids and gout with those of other research works is not possible currently as such an association was not investigated in the previous studies. In our study, among the group of amino acids showing positive relations with gout, Ile, Leu and Val are branched chain amino acids, and Phe and Trp are aromatic amino acids. PFAA, especially branched chain amino acids and aromatic amino acids are thought to be associated with lifestyle-related diseases [21]. Therefore, the association between lifestyle-related diseases and gout needs to be explored in future research works including a lager sample of patients with gouts.

### Limitations to the study findings

The present findings should be interpreted in the light of several possible study limitations. Firstly, both patients and controls included in this study were mainly of older age groups, and thus the generalizability of the current study findings is uncertain among younger populations. Secondly, a good percentage of the included subjects in both groups were under medications for diseases like hypertension, diabetes mellitus and dyslipidemia. There is a possibility that the results were confounded by those factors. However, we believe, this had little impact on the study results as the groups did not differ largely with respect to those characteristics. Thirdly, the small number of included subjects with gout is also a limitation of the present investigation. However, this was the natural outcome of the annual health examination data used in this study. Fourthly, we could not investigate the relationship of plasma uric acid level with amino acid levels in the study populations due to a limited number of available data on it. Lastly, the present study did not examine the mechanisms involved in the responses in amino acid profile caused by gout. Therefore, the exact reasons for the observed differences between the studies or the underlying mechanisms cannot be determined from the study findings.

## Conclusions

Taking all the findings of this study together, we conclude that significant alterations in plasma amino acid profile occurred in gout. Plasma levels of Ala, Ile, Leu, Phe, Trp, and Val had significant positive associations whereas Gly and Ser had significant inverse association with gout. The observed changes in PFAA profiles may have important implications for improving our understanding of pathophysiology and prevention of gout. Also, the specific variations in PFAA profiles and their association with gout as observed in our study might be useful in the diagnosis of this disease. The findings of this study need further confirmation in future large-scale studies involving a larger number of patients with gout.
